# Supplementary material for: Genetic associations with resistance to Meloidogyne enterolobii in guava (Psidium sp.) using cross-genera SNPs and comparative genomics to Eucalyptus highlight evolutionary conservation across the Myrtaceae
Source: PLoS One. 2022 Nov 2;17(11):e0273959. doi: 10.1371/journal.pone.0273959 (PMC9629644; doi:10.1371/journal.pone.0273959)
Supplement: Attachment — Submitted filename: Review_Guava.docx [file pone.0273959.s004.docx]

**Manuscript Title: Genetic associations with resistance to Meloidogyne enterolobii in guava (Psidium sp.) using cross-genera SNPs and comparative genomics to Eucalyptus highlight evolutionary conservation across the Myrtaceae**

**Manuscript Number: PONE-D-22-23102**

Overall comments:

The manuscript deals with the identification of genomic loci associated with resistance to Meloidogyne enterolobii in guava, the excellent fruit yielding tree species. It is an important research problem, where in several countries cultivate guava but the trees is routinely getting affected to nematodes. Marker assisted breeding is the suitable approach to produce pest resistant trees. The individuals were genotyped with EUChip60K containing Eucalyptus SNPs. Two SNP loci were found to be associated with nematode resistance. There are two publications available on the use of SNPs in Psidium and authors need to cite these references.

However, there is a complete and chromosome scale assembly of Psidium guajava with the coverage of about 95% has already been reported. The authors may utilise this genomic information and analyse SNP data generated to get the genomic coordinates of specific SNP regions identified from guava genome. Interpret the results based on the genomic regions of guava along with eucalypts.

The article is well written and reader friendly.

Specific comments:

| Line number | Author’s claim | Comments made |
| --- | --- | --- |
| 95-96 | while SNP markers are not yet available. | It is not correct. There are two publications available on the use of SNPs in Psidium. Authors need to quote these references   1. Grossi, L.L., Fernandes, M., Silva, M.A. et al. DArTseq-derived SNPs for the genus Psidium reveal the high diversity of native species. Tree Genetics & Genomes 17, 23 (2021). <https://doi.org/10.1007/s11295-021-01505-y> 2. Diaz‐Garcia, Luis, and José S. Padilla‐Ramírez. "Development of single nucleotide polymorphism markers and genetic diversity in guava (Psidium guajava L.)." Plants, People, Planet (2022). |
