# Supplementary material for: Genetic associations with resistance to Meloidogyne enterolobii in guava (Psidium sp.) using cross-genera SNPs and comparative genomics to Eucalyptus highlight evolutionary conservation across the Myrtaceae
Source: PLoS One. 2022 Nov 2;17(11):e0273959. doi: 10.1371/journal.pone.0273959 (PMC9629644; doi:10.1371/journal.pone.0273959)
Supplement: Attachment — Submitted filename: Responses_to_reviewers_PONE-D-22-23102.docx [file pone.0273959.s005.docx]

**PONE-D-22-23102 manuscript “Genetic associations with resistance to *Meloidogyne enterolobii* in guava (*Psidium* sp.*)* using cross-genera SNPs and comparative genomics to *Eucalyptus* highlight evolutionary conservation across the MyrtaceaePLoS One**

Below we respond to the reviewers’ comments

**Reviewer #1**

**REVIEWER COMMENT:** The manuscript deals with the identification of genomic loci associated with resistance to Meloidogyne enterolobii in guava, the excellent fruit yielding tree species. It is an important research problem, where in several countries cultivate guava but the trees is routinely getting affected to nematodes. Marker assisted breeding is the suitable approach to produce pest resistant trees. The individuals were genotyped with EUChip60K containing Eucalyptus SNPs. Two SNP loci were found to be associated with nematode resistance. There are two publications available on the use of SNPs in Psidium and authors need to cite these references.

**RESPONSE:** Thank you for pointing out those two papers. We have now included the two references in the manuscript. In the interest of the readership, we added a comment regarding the fact that these two SNP genotyping experiments were carried out using genotyping by sequencing methods based on genome complexity reduction by digestion with restriction enzymes. Although such methods do offer advantages as far as no upfront cost and simultaneous SNPs discovery and genotyping, they are well known to have low reproducibility across independent experiments and laboratories, and suffer with large proportions of missing data. As such, although SNPs are genotyped, they do not constitute a legacy genomic resource for future widespread and continuous use by the community, nor they allow consolidation of data across studies in the same manner as the chip-based SNPs data we have used in our work.

**REVIEWER COMMENT:** However, there is a complete and chromosome scale assembly of Psidium guajava with the coverage of about 95% has already been reported. The authors may utilize this genomic information and analyze SNP data generated to get the genomic coordinates of specific SNP regions identified from guava genome. Interpret the results based on the genomic regions of guava along with eucalypts.

**RESPONSE:** We are fully aware of the chromosome scale assembly of the Psidium guajava genome. In fact, we have cited that paper already in the introduction when presenting the available genomic resources for the species. As the reviewer might have noticed, in our work we have generated a de novo assembly of the Psidium guajava genome for a different Psidium guajava tree than the one for which the genome had been published. We did exactly what the reviewer is suggesting, i.e., we used the genome assembly we generated to transfer a large number of SNP probesets that had been developed for Eucalyptus to the Psidium genome at the nucleotide level providing the exact genomic coordinates. Please also note that we have deposited our genome assembly at DDBJ/ENA/GenBank under the accession JAGHRR000000000. The version described in this article is JAGHRR010000000. These resources were deposited under the BioProject ID PRJNA713343. The entire presentation of results, their interpretation and discussion were therefore based on a detailed mapping of the SNP probesets on the Psidium genome we have generated, which is fully syntenic to the previously published Psidium genome. Therefore, all the necessary genomic address information for the SNPs is made available to the readership for future use of the publicly available EUCHIP60K to further genotype Psidium genetic resources.

**Reviewer #2:**

**REVIEWER COMMENT:** In this manuscript an attempt has been made to identify the resistance gene for Meloidogyne enterolobii in Guava usingEUChip60K SNPs genotyping and de novo genome assembly of the Psidium guajava. The SNP genotyping could transfer 14,268 SNP probe sets from Eucalyptus to Psidium at the nucleotide level. Two SNPs (EuBR03s29615246 andEuBR03s30383415) on chromosome 3 in a pseudo-assembly of Psidium guajava genome built using a syntenic path approach with the Eucalyptus grandis genome were identified linked to resistance locus for RKN. This contribution should advance the development of new guava cultivars through the development of inexpensive SNP based assays to monitor the introgression of the P. guineense-derived resistance for RKN into guava cultivars and rootstocks.

**RESPONSE:** We thank the reviewer for the positive comment.

Sincerely,

***Dario Grattapaglia***

*Research Scientist*

*EMBRAPA Genetic Resources and Biotechnology*

*EPqB Final W5 Norte, Brasília, DF, 70770-917, Brazil*

***e-mail: dario.grattapaglia@embrapa.br***
